# Lung recruitment improves the efficacy of intubation-surfactant-extubation treatment for respiratory distress syndrome in preterm neonates, a randomized controlled trial

**DOI:** 10.1186/s12887-021-03096-y

**Published:** 2022-01-03

**Authors:** Yong Yang, Wenkang Yan, Minyi Ruan, Lan Zhang, Jinzhen Su, Haohui Deng, Minxu Li

**Affiliations:** 1 Department of Neonatology, Dongguan Maternal and Child Health Hospital, No. 99 Zhenxing Road, Dongcheng District, Dongguan, 523700 Guangdong Province China; 2 Department of Neonatology, Huizhou Central People’s Hospital, Huizhou, Guangdong Province China

**Keywords:** Lung recruitment, Respiratory distress syndrome, Intubation-surfactant-extubation (INSURE), Preterm neonates

## Abstract

**Background:**

Lung recruitment is a maneuver used to decrease the length of intubation in preterm neonates. This study aimed to compare the therapeutic efficacy of lung recruitment plus intubation-surfactant-extubation (INSURE) procedure and INSURE alone for the preterm neonates with respiratory distress syndrome.

**Methods:**

From 2017 to 2019, 184 preterm neonates (gestational age 24–32 weeks) with respiratory distress syndrome were enrolled and randomized into the lung recruitment group receiving lung recruitment (25 cm H_2_O, 15 s) plus INSURE and the control group receiving INSURE only. The primary outcome was the need for mechanical ventilation (MV) within 72 h after extubation. The secondary outcomes included duration of MV, noninvasive ventilation, total oxygen therapy, hospitalization time, and complications.

**Results:**

Compared to the control group, the lung recruitment group had a significantly lower proportion of preterm neonates requiring MV within 72 h after extubation (23% vs. 38%, *P* = 0.025) and pulmonary surfactant administration, as well as a shorter MV duration. There was no significant difference in the incidences of complications (all *P* > 0.05) and in-hospital mortality (2% vs. 4%, *P* = 0.4) between the lung recruitment group and control group. Multivariate logistic regression analysis demonstrated that the control group had a 2.17-time higher risk of requiring MV than the lung recruitment group (AOR: 2.17, 95% CI: 1.13–4.18; *P* = 0.021). Compared with infants with a normotensive mother, infants with a hypertensive mother have a 2.41-time higher risk of requiring MV (AOR: 2.41, 95% CI: 1.15–5.05; *P* = 0.020).

**Conclusion:**

Lung recruitment plus INSURE can reduce the need for MV within 72 h after extubation and did not increase the incidence of complications and mortality.

**Trial registration:**

Chinese Clinical Trial Registry ChiCTR1800020125, retrospectively registered on December 15, 2018.

## Background

Neonatal respiratory distress syndrome (RDS) is a leading cause of morbidity in preterm neonates, which is caused by pulmonary surfactant insufficiency and pulmonary structural immaturity [1]. The purpose of the management of RDS is to maximize survival and minimize potential adverse effects. According to the European Consensus Guidelines for the Management of RDS, preterm infants should receive Continuous positive airway pressure (CPAP) of at least 6 cm H_2_O via mask or nasal prongs to stabilize spontaneously breathing [2]. Persistently apneic or bradycardic infants should be given gentle positive pressure lung inflation with peak inspiratory pressure of 20–25 cm H_2_O [2]. The European Consensus Guideline also suggests that INSURE strategy should be considered for infants who failed in CPAP treatment [3]. The INSURE technique consists of an INtubation-SURfactant-Extubation procedure, which can reduce the need for mandatory ventilation (MV) and, the duration of respiratory support in preterm infants with RDS [4].

Lung recruitment is a maneuver with a peak pressure of 25–30cmH_2_O for 10–20 s [5]. It is used for preterm infants with respiratory failure and RDS to ensure early and effective creation of functional residual capacity (FRC) to prevent lung damage [6, 7]. Previous animal studies have shown that lung inflation can provide more stable tidal volume and uniform lung ventilation with improved FRC than intermittent ventilation [8, 9]. Clinical trials also demonstrate that preterm infants with RDS receiving lung recruitment at birth can decrease the need for MV [9–12]. Nevertheless, contradicting these findings, some trials reported that lung inflation has no superior effect [13, 14]. A review by Bruschettini et al. suggests that there is no evidence supporting that lung inflation is superior to IPPV in terms of MV requirement and other important respiratory outcomes [15]. On the other hand, in China, lung recruitment has not been extensively applied in clinical practice.

To further evaluate the therapeutic efficacy and safety of lung recruitment, this study aimed to compare the therapeutic efficacy of lung recruitment plus INSURE and INSURE alone in preterm neonates with RDS.

## Methods

### Participants and study design

**Fig. 1:**
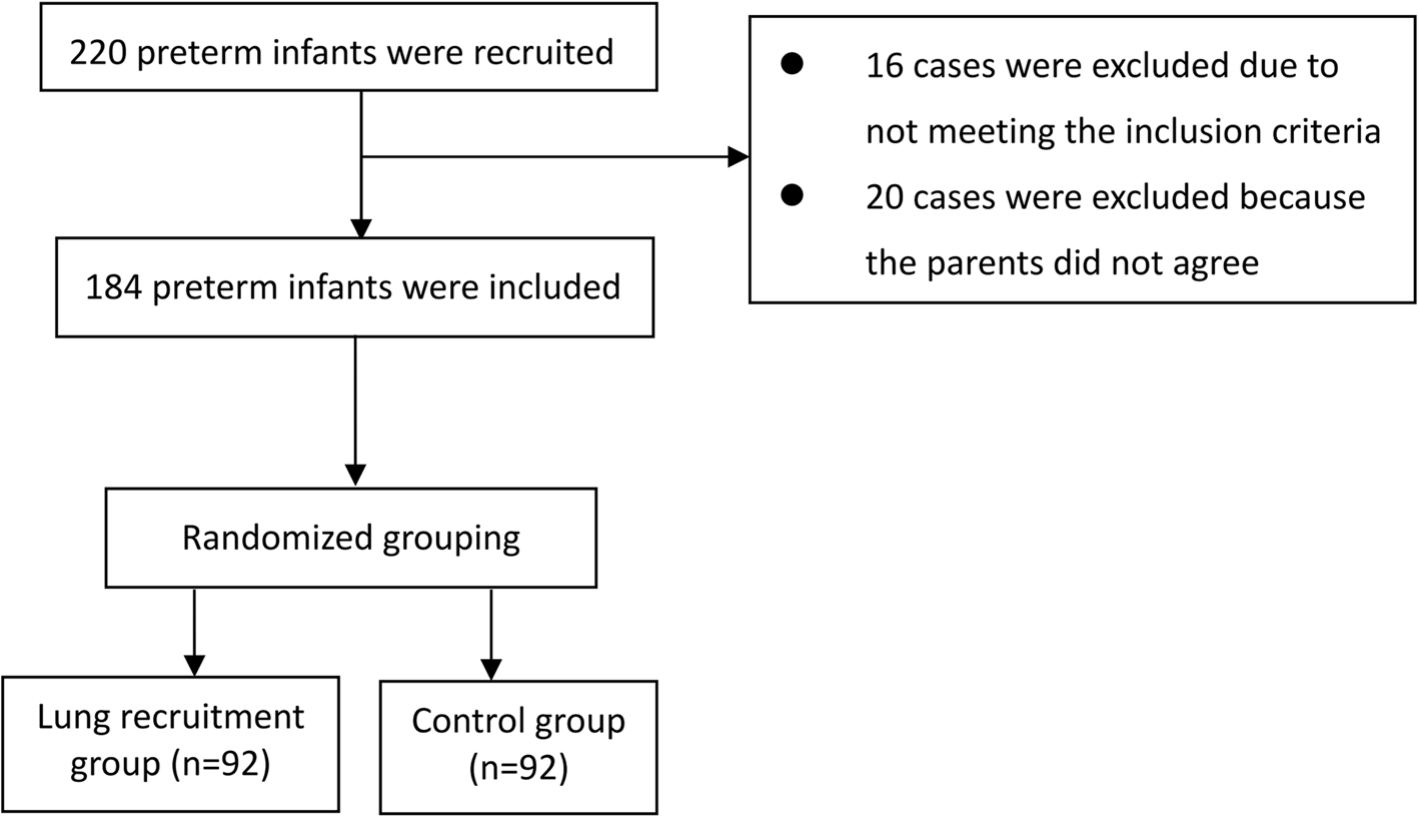
The trial flow diagram of this study

This was a prospective randomized controlled trial. Between 2017 and 2019, 184 preterm neonates with RDS treated in the Department of Neonatology in Dongguan Maternal and Child Health Hospital were enrolled and randomized into the lung recruitment group and the control group. This trial was registered at the Chinese Clinical Trial Registry (ChiCTR1800020125). The trial flow diagram was shown in Fig. 1.

Inclusion criteria were: 1) infants with gestational age from 24 to 32 weeks; 2) birth weight less than or equal to1800 g; 3) definite diagnosis of RDS by clinical syndrome and X-ray findings; 4) failure in CPAP treatment within 24 h after the parent’s consent. The infants with severe congenital malformation or required endotracheal intubation in the delivery room were excluded. This study was approved by the institutional review board of our hospital. Written informed consent was obtained from the parents. Our study adheres to CONSORT guidelines.

### Sample size calculation

We hypothesized that lung recruitment with INSURE could reduce the probability of mechanical ventilation after conventional INSURE therapy from 50 to 30%. The sample size was increased to 184 cases to achieve a power of 80% (a = 0.05).

### Randomization and treatment

The sequence numbers were kept in opaque sealed envelopes that were opened just before infants meet the inclusion criteria but not the exclusion criteria by a person not involving in the management of the infants. The infants in the lung recruitment group were intubated, and then received lung recruitment (25 cmH_2_O, 15 s) by a T-piece resuscitator and pulmonary surfactant (200 mg/Kg, as an early rescue therapy). The infants in the control group were intubated and then received pulmonary surfactant (200 mg/Kg). After the catheter was removed, the infants received CPAP therapy (6–8 cmH_2_O). Infants who failed in CPAP treatment after INSURE therapy were switched to mechanical ventilation with endotracheal intubation. A failure in CPAP treatment was defined as any of the following conditions: 1) inspired oxygen (FiO_2_) was equal to 0.4 or greater to maintain SpO_2_ at 88–94% for at least 30 min; 2) arterial blood gas analysis revealed respiratory acidosis: pCO_2_ > 65 mmHg (8.5 kPa), pH < 7.20; 3) more than 4 apneas per hour, or more than 2 severe apneas requiring positive pressure ventilation with a bag valve mask. The ventilator conditions were set as follows: PIP20-22cmH_2_O, PEEP5-6cmH_2_O, RR30–40 beats/min to maintain VT4–6 ml/kg pCO_2_ 40–60 mmHg. Mechanical ventilation can be stopped when the infants reached the following conditions: FiO_2_ ≤ 0.3, MAP≤8 cmH_2_O, pCO_2_ ≤ 55 mmHg, pH ≥7.25, SpO_2_ ≥ 88% for more than 8 h, and hemodynamics were stable. After extubation, the infants received CPAP (6–8 cmH_2_O). In the control group, all infants also received INSURE and endotracheal intubation, but not lung recruitment. The other procedures were the same as the intervention group. All infants meeting the CPAP failure criteria again within 12–24 h after extubation were given a second dose of surfactant (100 mg/kg) by the same methods in both the lung recruitment or control group.

### Outcome measures

The following data were recorded: durations of MV, noninvasive ventilation, total oxygen therapy, and hospitalization and the complications, including pneumothorax, intraventricular hemorrhage (IVH), necrotizing enterocolitis (NEC), retinopathy of prematurity (ROP), patent ductus arteriosus (PDA), persistent pulmonary hypertension (PPHN), bronchopulmonary dysplasia (BPD) and death. The IVH was defined as a spectrum of hemorrhage brain injury most typically occurring in the first week of life in very preterm babies according to the NIH Consensus Development Conference [16]. BPD was defined according to the oxygen requirement as follows: Mild BPD- a need for supplemental oxygen (O_2_) for ≥28 days but not at 36 weeks’ postmenstrual age (PMA) or discharge; moderate BPD- a need for supplemental O_2_ for 28 days plus treatment with< 30% O_2_ at 36 weeks’ PMA; severe BPD- a need for supplemental O_2_ for ≥28 days plus ≥30% O_2_ and/or positive pressure at 36 weeks’ PMA [17]. Meanwhile, blood samples were collected from the radial artery for blood gas analysis before extubation, as well as 1, 6, 12, 24, 48, and 72 h after extubation.

### Statistical analysis

Statistical data were analyzed by using Student’s t-test for parametric and the Mann–Whitney U test for non-parametric continuous variables; and χ2 test or Fisher’s exact test (if any expected value < 5 was found) for categorical variables. Repeated measurement data were used in the General Linear Model. Univariate and multivariate logistic regression was used to investigate risk factors of requiring MV within 72 h after extubation. The variables significant in univariate results would be entered into a multivariate model, and the variables significant in multivariate results would be considered as an independent factor associated with the primary outcome. The statistical significance level was set at a *P*-value < 0.05. All analyses were performed using IBM SPSS Version 20 (SPSS Statistics V20, IBM Corporation, Somers, New York).

## Results

### Patients’ demographic and clinical characteristics

A total of 184 preterm neonates with RDS were randomized into the lung recruitment group which received lung recruitment plus INSURE and the control group which received INSURE only (*n* = 92 for each group). Patients’ demographic and clinical characteristics were summarized in Table 1. Both the lung recruitment group and the control group had a mean gestational age of around 29.7 weeks and a mean birth weight of around 1.30 kg. There was no significant difference in infants at birth including gestational age, birth weight, gender, and rate of cesarean section (all *P* > 0.05). There was no significant difference in the mother’s mean age between the lung recruitment group and the control group (29.74 ± 5.19 vs. 30.71 ± 5.27 years, *P* = 0.211). The mothers of neonates in the control group had a higher incidence of gestational diabetes mellitus than those in the lung recruitment group (*P* = 0.005, Table 1).

**Table 1:** Patients’ demographic and clinical characteristics

	lung recruitment group (*n* = 92)	Control group (*n* = 92)	t/χ2	P
Gestational age, mean (SD), wk	29.75 ± 1.86	29.69 ± 1.66	0.218	0.828
Birth weight, mean (SD), kg	1.32 ± 0.28	1.29 ± 0.25	0.719	0.473
Gender,boy, n(%)	55 (60)	60 (65)	0.580	0.446
Cesarean section, n(%)	56 (61)	65 (71)	1.955	0.162
**Mothers’ characteristics**				
Mother’s age, mean (SD), year	29.74 ± 5.19	30.71 ± 5.27	1.254	0.211
Antenatal steroids, n(%)	54 (59)	48 (52)	0.792	0.374
gestational diabetes mellitus, n(%)	8 (9)	22 (24)	7.806	0.005
amniotic fluid pollution, n(%)	7 (8)	10 (11)	0.583	0.445
intrauterine infection, n(%)	29 (32)	34 (37)	0.603	0.437
Hypertension disorders, n(%)	21 (23)	19 (21)	0.128	0.721
premature rupture of membranes, n(%)	16 (17)	20 (22)	0.553	0.457
Asphyxia, n(%)	18 (20)	15 (16)	0.332	0.564
Apgar,1 min,median (minimum,maximum)	9 (3,10)	10 (2,10)	0.703	0.483
Apgar,5 min,median (minimum,maximum)	10 (7,10)	10 (6,10)	0.560	0.576
Apgar,10 min,median (minimum,maximum)	10 (8,10)	10 (7,10)	0.379	0.705

### Therapeutic outcomes and complications

Therapeutic outcomes were compared between the two groups. As shown in Table 2, the lung recruitment group had significantly fewer preterm neonates requiring MV within 72 h after extubation as compared with the control group (23% vs. 38%, *P* = 0.025). In addition, the MV duration, the averaging times of using pulmonary surfactant and the maximum FiO_2_ were significantly reduced in the lung recruitment group than in the control group (all *P* < 0.05, Table 2). Nevertheless, no other secondary outcomes were significantly different between the two groups (all *P* > 0.05, Table 2).

**Table 2:** The comparison of therapeutic outcomes between the two groups

	lung recruitment group (*n* = 92)	Control group (*n* = 92)	Z/χ2	P
Primary outcome, n(%)				
MV within72 h after extubation	21 (23)	35 (38)	5.031	0.025
Second outcomes				
Mechanical ventilation duration,^a^,d	0 (0–3.8)	1.5 (0–9)	2.73	0.006
Noninvasive ventilation duration, ^a^,d	11.6 (6–28)	13.8 (7–25.4)	0.426	0.670
Total oxygen therapy duration, ^a^,d	22.6 (9.7–35)	26.5 (14–39)	0.804	0.421
Hospitalization, ^a^,d	45 (32–62)	47 (38–67)	0.869	0.385
Pulmonary surfactant operate duration, ^a^,h	3 (1.5–5)	3 (2–6)	1.072	0.284
Averaging times of using pulmonary surfactant ^b^	1.05 ± 0.23	1.17 ± 0.44	2.199	0.028
maximum FiO_2_^b^	0.32 ± 0.05	0.35 ± 0.09	0.869	0.004

^a^: median (P25, P75), ^b^: mean ± SD

**Fig. 2:**
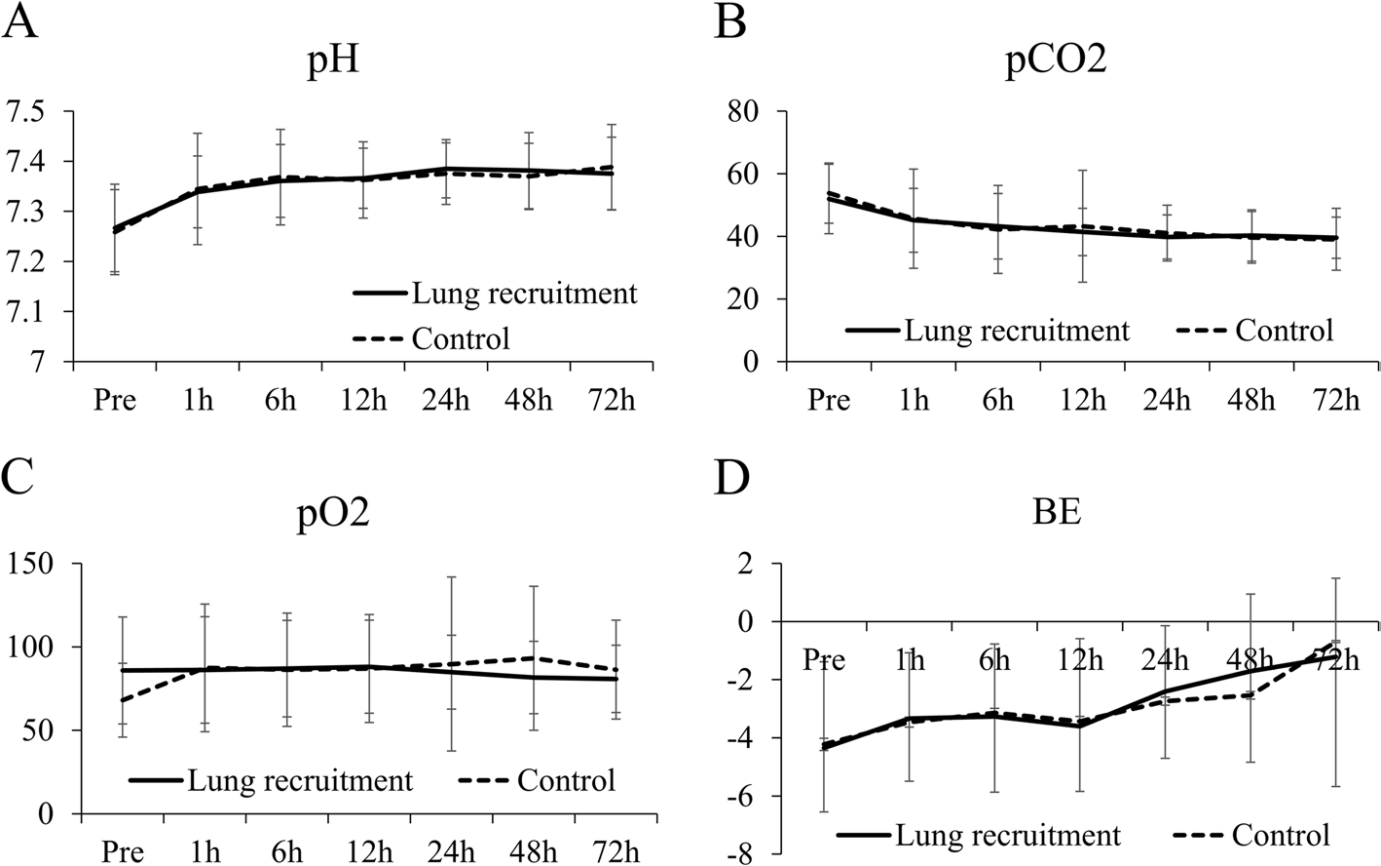
The change of pH (**A**), pCO_2_ (**B**), pO_2_ (**C**), and BE (**D**) before extubation (pre) to 72 h after extubation

The 4 parameters of blood gas were compared between the two groups, including pH value (Fig. 2A), pCO_2_ (Fig. 2B), pO_2_ (Fig. 2C), and Base Excess (BE, Fig. 2D). No significant difference was observed between the two groups (P > 0.05).

As shown in Table 3, there was no significant difference in the incidences of complications between the lung recruitment and control groups (all P > 0.05), including BPD (33% vs. 40%), IVH (9% vs. 19%), pneumothorax (2% vs. 3%), NEC (11% vs. 16%), PDA (23% vs. 26%), ROP (21% vs. 15%), and PPHN (2% vs. 4%). Two cases (2%) in the lung recruitment group and 4 (4%) cases in the control group died because their parents gave up treatment due to financial considerations or possible serious complications (*P* = 0.4).

**Table 3:** The comparison of complications between the two groups

	lung recruitment group (*n* = 92)	Control group (*n* = 92)	χ2	P
BPD, n(%)	30 (33)	37 (40)	1.15	0.284
mild BPD	17 (57)	28 (76)	2.714	0.099
moderate-severe BPD	13 (43)	9 (24)	0.6498	0.420
IVH, n(%)	8 (9)	17 (19)	3.749	0.053
pneumothorax, n(%)	2 (2)	3 (3)	0.000	1.000
NEC, n(%)	10 (11)	15 (16)	1.157	0.282
PDA, n(%)	21 (23)	24 (26)	0.265	0.607
ROP, n(%)	19 (21)	14 (15)	0.923	0.337
PPHN, n(%)	2 (2)	4 (4)	0.689	0.406
Death, n(%)	2 (2)	4 (4)	0.689	0.406

*BPD* bronchopulmonary dysplasia, *IVH* intraventricular hemorrhage, *NEC* necrotizing enterocolitis, *PDA* symptomatic patent ductus arteriosus, *ROP* retinopathy of prematurity, *PPHN* persistent pulmonary hypertension

### Independent factors associated with the need for MV within 72 h after extubation

To further investigate the risk factor of requiring MV within 72 h after extubation, univariate, and multivariate logistic regression models were performed. As shown in Table 4, the control group had a 2.17-time higher risk of requiring MV than the lung recruitment group (adjusted odds ratio [AOR]: 2.17, 95% CI: 1.13–4.18; *P* = 0.021). Compared with infants with a normotensive mother, infants with a hypertensive mother have a 2.41-time higher risk of requiring MV (AOR: 2.41, 95% CI: 1.15–5.05; *P* = 0.020).

**Table 4:** Logistic regression analysis of risk factors associated with the need for mechanical ventilation within 72 h after extubation

Parameters	Univariate		Multivariate	
	OR (95% CI)	P	AOR (95% CI)	P
Group				
lung recruitment	ref.	–	ref.	–
Control	2.08 (1.09 to 3.95)	0.026	2.17 (1.13 to 4.18)	0.021
Gestational age, week	0.84 (0.70 to 1.00)	0.055		
Birth weight, kg	0.51 (0.16 to 1.66)	0.263		
Gender of newborn				
Male	ref.	–		
Female	0.72 (0.37 to 1.39)	0.322		
Delivery method				
NSD	ref.	–		
CS	1.14 (0.59 to 2.23)	0.692		
Mother				
Antenatal steroids, yes	0.59 (0.31 to 1.12)	0.105		
Gestational diabetes mellitus, yes	0.98 (0.42 to 2.29)	0.955		
Amniotic fluid pollution, yes	0.95 (0.32 to 2.83)	0.923		
Intrauterine infection, yes	1.53 (0.80 to 2.94)	0.198		
Hypertension, yes	2.28 (1.11 to 4.71)	0.026	2.41 (1.15 to 5.05)	0.020
Premature rupture of membranes, yes	0.85 (0.38 to 1.91)	0.699		
Asphyxia				
No	ref.	–		
Yes	1.91 (0.88 to 4.16)	0.102		
Apgar - 1 min	0.87 (0.74 to 1.02)	0.076		
Apgar - 5 min	0.80 (0.55 to 1.18)	0.261		
Apgar - 10 min	0.61 (0.36 to 1.03)	0.066		

*NSD* normal spontaneous delivery, *CS* cesarean section, *OR* odds ratio, *AOR*, adjusted odds ratio

## Discussion

In this study, we compared the therapeutic efficacy of lung recruitment plus INSURE and INSURE alone for the preterm neonates with RDS. The results showed that compared to the control group, the lung recruitment group had a significantly lower proportion of preterm neonates requiring MV within 72 h after extubation (23% vs. 38%, *P* = 0.025) and pulmonary surfactant administration, as well as a shorter MV duration. Nevertheless, there were no significant differences in other secondary outcomes, 4 parameters of blood gas, and the incidence of complications between the two groups. Multivariate logistic regression analysis demonstrated that the control group had a 2.17-time higher risk of requiring MV than the lung recruitment group (AOR: 2.17, 95% CI: 1.13–4.18; *P* = 0.021). Compared with infants with a normotensive mother, infants with a hypertensive mother have a 2.41-time higher risk of requiring MV (AOR: 2.41, 95% CI: 1.15–5.05; *P* = 0.020).

Lista et al. conducted a clinical trial in which preterm infants with RDS treated with sustained lung inflation (25 cm H_2_O, sustained for 15 s) at birth and concluded that preterm infants with RDS received sustained lung inflation at birth may decrease the need for MV and did not induce adverse effects as compared with a historical control group [18]. Therefore, the condition of 25 cm H_2_O for 15 s was used in this study. Consistent with Lista et al.’s observation, our results suggested that lung recruitment can effectively reduce the need for MV but did not increase the adverse effects. In addition, both MV duration and the number of pulmonary surfactant administration were significantly reduced in the lung recruitment group as compared with the control group, which were in line with previous findings [11, 12, 18–20]. The lung recruitment technique might positively affect the clearance of lung fluid and allows a more even distribution of air throughout the lungs, thus facilitating the formation of FRC [21]. Therefore, the beneficial effects may be attributed to lung recruitment, subsequent FRC achievement and inflation-induced alveolar expansion.

BPD is a major complication of preterm birth [22] and has a complicated pathogenic mechanism. Immature lung development, acute lung injury, and abnormal repairment after injury are key points leading to BPD [23]. One of the most important pathogenic factors of BPD is ventilator-induced lung injury [24]. In this study, the incidence of BPD was lower in the lung recruitment group than in the control group (33% VS 40%), but the difference did not reach statistical significance. The incidence of different severe BPDs was also not significantly different between the two groups. This result suggested that lung recruitment did not increase the incidence of BPD. In this study, lung recruitment did not increase the incidences of adverse effects, including IVH, NEC, PDA, ROP, which is in agreement with previous studies [13, 14, 18, 25].

In this study, neonates of hypertensive mothers had a higher risk of the need for MV within 72 h after extubation than those of normotensive mothers. It has been shown that gestational hypertension can promote maternal production of soluble fms-like tyrosine kinase-1 (sFlt-1), an anti-angiogenic factor that can block vascular endothelial growth factor (VEGF) signaling [26, 27]. Since VEGF signaling is essential for the growth of pulmonary blood vessels and the production of surfactants, sFlt-1 may lead to increased incidence and severity of RDS in preterm neonates with hypertensive mothers. Lung recruitment seemed did not help these patients, and the underlying mechanism is needed to be further investigated.

In this study, two cases had pneumothorax in the lung recruitment group and three cases had pneumothorax in the control group, suggesting that lung recruitment did not increase the risk of pneumothorax. This result is consistent with previous reports [10, 14, 15, 25]. By contrast, Lista et al. have reported that patients with lung inflation treatment have a 4.57-time high risk of pneumothorax than the control patients [11]. The discrepancy might be attributed to different study subjects, and the effect of lung recruitment on pneumothorax should be further investigated.

In this study, no difference in mortality was found between the two groups. Two cases of death in the lung recruitment group because their parents gave up treatment rather than pneumothorax or other severe complications. This result suggested lung recruitment did not increase mortality. The comparison in the blood gas parameters between the two groups showed no significant difference, suggesting that lung recruitment did not impact the circulation and the rate of acidosis. This may be due to the comprehensive influence of ventilation improvement.

There are still some limitations to this study. First, the study was not a double-blind design. The staffs performing the study also cared for the infants later, which might affect the outcomes. We tried to minimize this bias by strictly following the trial protocol during the whole trial. In addition, this was a single-center trial and the sample size was still relatively small. In the future, a large multicenter trial should be conducted to validate the findings of this study.

## Conclusion

Our study found that lung recruitment plus INSURE can effectively reduce the need for MV within 72 h after extubation and did not increase the incidence of complications and mortality.

## Data Availability

The datasets used and/or analysed during the current study are available from the corresponding author on reasonable request.

## Abbreviations

RDS: Respiratory distress syndrome
CPAP: Continuous positive airway pressure
MV: Mandatory ventilation
FRC: Functional residual capacity
IVH: Intraventricular hemorrhage
NEC: Necrotizing enterocolitis
ROP: Retinopathy of prematurity
PDA: Patent ductus arteriosus
PPHN: Persistent pulmonary hypertension
BPD: Bronchopulmonary dysplasia
PMA: Postmenstrual age
